# Introduction to the PLOS ONE collection on ‘Understanding and preventing suicide: Towards novel and inclusive approaches’

**DOI:** 10.1371/journal.pone.0264984

**Published:** 2022-03-10

**Authors:** Jo Robinson, Kairi Kolves, Merike Sisask

**Affiliations:** 1 Orygen, Parkville, Victoria, Australia; 2 Centre for Youth Mental Health, University of Melbourne, Parkville, Victoria, Australia; 3 Australian Institute for Suicide Research and Prevention, WHO Collaborating Centre for Research and Training in Suicide Prevention, School of Applied Psychology, Griffith University, Brisbane, Australia; 4 Tallinn University, School of Governance, Law and Society, Tallinn, Estonia; 5 Estonian-Swedish Mental Health and Suicidology Institute (ERSI), Tallinn, Estonia; Public Library of Science, UNITED STATES

## Abstract

More than 700,000 people lose their lives to suicide each year and evidence suggests that the current COVID-19 pandemic is leading to increases in risk factors for suicide and suicide-related behaviour, in particular among young people. It is widely documented that some sectors of the population are over-represented in the suicide statistics. It is also well established that the pathways that lead someone to a suicidal crisis are complex and differ across regions and sectors of the population; as such a multi-faceted approach to prevention is required. Many of us would also argue that novel approaches, that combine broad population-based strategies with individual interventions, and approaches that capitalise on new technologies and methodologies are also required. For these reasons, when bringing together this collection, we deliberately sought studies that focused upon those groups who are over-represented in the suicide statistics yet under-represented in research. We also called for studies that reported on novel approaches to suicide prevention and for studies that reflected the voices of people with lived experience of suicide, also often unheard in research efforts.

## Introduction

Suicide is a significant public health problem around the world. According to recent data from the World Health Organization more than 700,000 people lose their lives to suicide each year [1]; many more people live with suicidal ideation and behaviour. Globally more suicides occur in males than females and the majority occur in low-middle-income countries where most of the world’s population live. Over half of the world’s suicides occur in people under the age of 50. Suicide is the fourth leading cause of death among adolescents and the numbers of deaths in this age group are relatively similar among both males and females. However, important regional differences exist. For example, females in low-middle-income countries have much higher suicide rates than females in high-income countries whilst the reverse is the case for males [1].

There are also a number of sub-groups of the population that are over-represented in the suicide statistics, such as people who identify as LGBTQIA+, Indigenous people, and people with mental illness [1–3].

Over the past 20 years the global rate of suicide has decreased, however, as above, this is not uniformly the case. Rates have increased in the Region of the Americas [1] and parts of Asia [4] and in many parts of the world they are steadily increasing amongst young people, in particular young females [5–9], with suicide representing the leading cause of death among young people in some countries including Australia and New Zealand [10, 11].

As such, suicide prevention approaches need to be regionally targeted, age appropriate and culturally sensitive.

### Current context

Whilst it is encouraging to see overall suicide rates decreasing, concerns currently exist regarding the potential impacts of the COVID-19 pandemic on mental health and suicide risk.

Several systematic reviews and meta-analyses have identified an association between the pandemic and high levels of psychological distress, depression and anxiety [12, 13] and there are concerns that this will have a knock-on effect on rates of both suicide and suicide-related behaviour [14]. At the time of writing, the most recent reports suggest that suicide rates have not increased at population-level [15]. However, this was not uniformly the case with some countries reporting increases in rates of suicide in young people, and particularly young females [16, 17] among whom (as noted above) rates were already on the rise. It has also been argued that some sectors of the population (arguably young people and those already at elevated risk) will be disproportionately impacted by the health and socio-economic impacts of the pandemic [18] thereby potentially placing them at greater risk of suicide in the future.

This does not mean that a global increase in rates of suicide is inevitable, but it does mean that we need to increase our efforts to identify and deliver effective suicide prevention strategies, including those that address the health, socio-economic and cultural determinants of risk.

As such this collection is timely. When we launched the call for papers for this collection we invited papers that focused upon those groups who are over-represented in the suicide statistics yet under-represented in research, and those that reported on novel approaches to suicide prevention. We also called for studies that reflected the voices of people with lived experience of suicide–again often unheard in research efforts.

### Collection overview

Here we present a collection of papers that together represent the complexity of suicide. We have studies from a number of countries including those that examine suicide prevention approaches in low-middle-income countries (e.g., Bangladesh, Brazil, Ethiopia, India, Nigeria). We also have studies that report on efforts to reduce suicide in different population groups, including young people, people who identify as same-sex attracted or gender diverse, and Aboriginal and Torres Strait Islander people. We have papers that report on studies conducted in a range of different settings including schools, prisons, and clinical settings, and we also have studies that have used novel approaches to tackle the problem of suicide and its sequelae, including several that have examined the potential of machine learning models for more accurate prediction of suicide risk and some that examine the impacts of social media-based interventions.

Further, the collection not only brings together studies that focus on individual approaches to suicide prevention but also those that tackled some of the larger social determinants of suicide-related behaviour such as socio-economic and employment factors that, as noted above are critical in the current landscape. For example, perceived low economic status was shown to increase the risk of persistent suicidality in sexual minority in six European countries [19]. Similarly, an aggregated level analysis from Spain showed that economic instability introduced by specific reforms liberalising the use of fixed-term contracts (i.e., increasing temporary contracts and reducing the number of days in employment), led to an increase in suicides [20].

#### Vulnerable populations

The majority of the studies focused on children and adolescents in different settings and only a few directly targeted other age groups specifically (e.g., older people, married adults).

The vulnerability of females emerged in several papers. For example, a South Korean study examining the impact of celebrity suicides found girls to be more negatively affected than boys [21] and a study from the Netherlands identified that girls began communicating about suicide at a younger age and their communication was more explicitly suicide-related than their male counterparts. However, much of their communication appeared to be focused on coping and seeking support from others, which may be taken positively [22]. Finally, the study by Rafael and colleagues identified particularly high prevalence of suicidal behaviour among Brazilian trans women, linked to violence and poor mental health [23].

Indeed, this collection clearly identified higher risk of suicidality among specific vulnerable groups, including sexual minorities, Indigenous people and refugees. For example, the study by Gebremeskel found the prevalence of suicidal ideation to be high among Eritrean refugees in Tigray, Ethiopia, in particular amongst those with a previous history of trauma, post-traumatic stress disorder, and a family history of mental illness [24]. Finally, the study by Ritland and colleagues identified a relationship between child apprehension and suicide attempt among a cohort of young Indigenous women in Canada who had been impacted by substance use [25].

It is well established that people from Indigenous backgrounds are frequently over-represented in the suicide statistics so it was heartening to also see studies that focused on preventative measures. For example, the study by Armstrong and colleagues examined the impact of culturally appropriate guidelines on the delivery of mental health first aid among Australian Aboriginal and Torres Strait Islander people with promising results [26].

#### Novel approaches

The papers included here reported on studies that used a variety of different study designs, and although the majority used quantitative methods (e.g., time-series, case-control, cohort and other designs) some employed qualitative methods (e.g., grounded theory, interpretative phenomenological analysis). In addition, a number of studies utilized rather novel statistical methods, which can be applied to large datasets using automated or routine data collection (e.g., social media platforms or hospital administrative systems). For example, an Australian study of Victorian ambulance data used latent trajectory modelling to identify different trajectories of suicide attempt repeaters by the lethality of methods used [27]. Another study from Australia examining data from mental health services used machine learning algorithms to predict repetition of self-harm in young people [28]. These novel methods enable a deeper understanding of help-seeking and intentional self-harm, which in turn (it is hoped) will facilitate an improved, and more personalised, approach to clinical assessment and care.

There were also a number of studies that focused on various social media platforms, including YouTube and Reddit, some of which also adopted novel approaches [29–31]. The role of the media, and in particular social media, has generated much interest in recent years with a number of studies examining both the potential harms and benefits associated with social media when it comes to suicide prevention [32, 33]. However, the pandemic and the associated lockdowns has meant that people have become increasingly socially isolated and as a result there has been a significant rise in the use of social media [34]. This is likely to be particularly the case amongst young people who were already heavy users of social media [35]; it is also likely to include involve use for both social and health-related purposes. For this reason, it was both encouraging and timely to see these studies included here.

#### The voice of lived experience

Finally, as noted above we were also keen for the voices of people with lived experience to be reflected in the collection. The importance of involving people with lived experience in research is being increasingly recognised [36]. Often termed public and patient involvement (or PPI), this can include varying levels of involvement ranging from passive (e.g., where individuals participate as research subjects) to more active involvement (e.g., membership of a steering committee), right through to user-led research where every stage of the research is driven by people with lived experience. Indeed, many funding bodies and journals now require investigators to demonstrate the ways in which consumers have been involved in both the planning and conduct of research, and distinct benefits exist, including increased relevance of the research and improved recruitment and retention rates [37, 38]. However, barriers, such as lack of knowledge or confidence on the part of researchers as well as resource limitations e.g., funding, training, and the capacity to provide adequate support also exist [37–39]. As as result, unfortunately suicide prevention research that actively engages consumers in a meaningful way remains rare [40]. In this collection whilst most of the studies would have included consumers as passive participants, a few also specifically sought to examine the perspectives of those with lived experience either of feeling suicidal themselves [41] or of having been bereaved by suicide [42], and the study by LaSala et al reported having partnered with young consumers on the development of the intervention being tested [31]. That said disappointingly, but perhaps unsurprisingly, none reported including consumers as research partners and to the best of our knowledge no studies were user-led.

### Closing remarks

The papers in this collection represent a broad range of studies that examine suicide risk and prevention across the globe. Together the studies report on suicide prevention efforts in a number of different population groups, including those who may be particularly disadvantaged by the pandemic (e.g., young people and people in low-middle-income countries) and they cover the broad spectrum of population- and individually-based approaches. They also report on a number of novel approaches to suicide prevention, which we believe are much needed if we are to improve our risk detection, assessment and treatment capabilities. Finally, these studies go some way towards enhancing our understanding of people with lived experience of suicide, from the perspectives of individuals who have experienced a suicidal crisis, those who have been bereaved by suicide, and those who have been exposed to suicide through the work that they do [43]. We hope that this collection not only conveys the complexity of suicide but also highlights innovative approaches that can help to inform future preventative efforts.
